# Most photorespiratory genes are preferentially expressed in the bundle sheath cells of the C_4_ grass *Sorghum bicolor*

**DOI:** 10.1093/jxb/erw041

**Published:** 2016-03-14

**Authors:** Florian Döring, Monika Streubel, Andrea Bräutigam, Udo Gowik

**Affiliations:** ^1^Institute of Plant Molecular and Developmental Biology, Universitätsstrasse 1, Heinrich-Heine-University, D-40225 Düsseldorf, Germany; ^2^Institute of Plant Biochemistry, Universitätsstrasse 1, Heinrich-Heine-University, D-40225 Düsseldorf, Germany; ^3^Cluster of Excellence on Plant Sciences (CEPLAS)‘From Complex Traits towards Synthetic Modules’, D-40225 Düsseldorf, Germany

**Keywords:** C_4_ photosynthesis, CO_2_ fixation, differential gene expression, evolution, photorespiration, *Sorghum bicolor*.

## Abstract

One of the hallmarks of C_4_ plants is the division of labor between two different photosynthetic cell types, the mesophyll and the bundle sheath cells. C_4_ plants are of polyphyletic origin and, during the evolution of C_4_ photosynthesis, the expression of thousands of genes was altered and many genes acquired a cell type-specific or preferential expression pattern. Several lines of evidence, including computational modeling and physiological and phylogenetic analyses, indicate that alterations in the expression of a key photorespiration-related gene, encoding the glycine decarboxylase P subunit, was an early and important step during C_4_ evolution. Restricting the expression of this gene to the bundle sheath led to the establishment of a photorespiratory CO_2_ pump. We were interested in whether the expression of genes related to photorespiration remains bundle sheath specific in a fully optimized C_4_ species. Therefore we analyzed the expression of photorespiratory and C_4_ cycle genes using RNA *in situ* hybridization and transcriptome analysis of isolated mesophyll and bundle sheath cells in the C_4_ grass *Sorghum bicolor*. It turns out that the C_4_ metabolism of *Sorghum* is based solely on the NADP-dependent malic enzyme pathway. The majority of photorespiratory gene expression, with some important exceptions, is restricted to the bundle sheath.

## Highlight

Photorespiratory genes are expressed tissue-specific in the leaves of the C_4_ grass *Sorghum bicolor*. Most but not all of them are confined to the bundle sheath cells.

## Introduction

C_4_ plants evolved multiple times from C_3_ ancestors. The C_4_ photosynthetic pathway leads to concentration of CO_2_ around the main carboxylating enzyme ribulose-1,5-bisphosphate carboxylase/oxygenase (RubisCO). This is achieved by a set of anatomical and biochemical modifications to the original C_3_ pathway (Hatch, 1987). In the presence of high CO_2_ concentrations, the oxygenase activity of RubisCO, which always competes with the carboxylation reaction, is effectively suppressed and hence photorespiration is strongly reduced in C_4_ plants (Hatch, 1987). Photorespiration occurs when O_2_ is used by RubisCO, which leads to the production of 2-phosphoglycolate (2-PG), a compound which is toxic for the plant cell and which needs to be detoxified (Anderson, 1971). Photorespiration takes place in chloroplasts, peroxisomes, and mitochondria. Throughout the regeneration of phosphoglycerate from phosphoglycolate, previously fixed CO_2_ is lost and additional energy and reduction equivalents are needed. Hence photorespiration can reduce the efficiency of photosynthesis in C_3_ species by up to 30% (Ogren, 1984; Bauwe *et al.*, 2010; Raines, 2011; Fernie *et al.*, 2013). Therefore, C_4_ photosynthesis can be of great advantage in conditions that promote photorespiration, such as hot, arid, and saline environments, in which plants have to close their stomata in order to avoid water loss through transpiration but which in consequence hinders the uptake of CO_2_ (Sage, 2004). C_4_ plants can keep their stomata closed for a longer time, because the CO_2_ pump facilitates high rates of photosynthesis even under low CO_2_ concentrations in the intercellular air space of the leaf and therefore minimizes water loss.

Leaves of C_4_ plants show anatomical differences compared with those of C_3_ plants. The vascular bundles are surrounded by organelle-rich bundle sheath cells, which, in turn, are surrounded by mostly one layer of mesophyll cells. This leads to a wreath-like appearance, which is termed Kranz anatomy (Haberlandt, 1904; Laetsch, 1974). In C_4_ leaves, bundle sheath cells are enlarged and the interveinal distance is reduced (Dengler and Nelson, 1999). To allow the efficient interchange of metabolites between mesophyll and bundle sheath cells, both cell types are connected through numerous plasmodesmata (Botha, 1992).

In most species, C_4_ photosynthesis largely depends on the division of labor between mesophyll and bundle sheath cells, in which the CO_2_ assimilatory enzymes are compartmentalized. The C_4_ pathway begins with the conversion of CO_2_ to bicarbonate by carbonic anhydrase (CA) in the cytosol of mesophyll cells and the subsequent fixation into the C_4_ acid oxaloacetate by phosphoenolpyruvate carboxylase (PEPC) with the 3-carbon compound phosphoenolpyruvate (PEP) as CO_2_ acceptor. Afterwards, oxaloacetate is either reduced to malate or transaminated to aspartate, which is transported to the bundle sheath cells. There, CO_2_ is released by decarboxylation of the C_4_ compounds through a decarboxylating enzyme, either an NADP-dependent malic enzyme (NADP-ME), an NAD-dependent malic enzyme (NAD-ME), a PEP-carboxykinase (PEP-CK), or, as shown recently, a combination of these (Furbank, 2011; Y. Wang *et al.*, 2014). The released CO_2_ is immediately refixed by RubisCO and enters the Calvin–Benson cycle. Less RubisCO is needed compared with C_3_ plants as it works more efficiently under these conditions (Long, 1999). This results in a better nitrogen use efficiency of C_4_ plants, since RubisCO is by far the most abundant protein in the leaves of higher plants (Long, 1999). Pyruvate, the other product of the decarboxylation, is transferred to the mesophyll cells where PEP is regenerated by pyruvate phosphate dikinase (PPDK).

C_4_ photosynthesis has evolved at least 66 times independently from the original C_3_ pathway (Sage *et al.*, 2011, 2012). To better understand the changes underlying the evolution of C_4_ on the gene level, in recent years several studies aimed at creating transcriptome atlases of total leaf RNA of various pairs of closely related C_4_ and C_3_ species (Bräutigam *et al.*, 2011, 2014; Gowik *et al.*, 2011; Mallmann *et al.*, 2014). The development of C_3_ and C_4_ leaves was studied by analyzing the gene expression in different developmental stages of dicot leaves and the developmental gradients found in the leaves of C_3_ and C_4_ grasses (Li *et al.*, 2010; Pick *et al.*, 2011; Kulahoglu *et al.*, 2014; L. Wang *et al.*, 2014; Ding *et al.*, 2015). The co-ordination of the two different cell types was analyzed using mesophyll and bundle sheath transcriptomes of the C_4_ grasses maize and *Setaria viridis* (Li *et al.*, 2010; Chang *et al.*, 2012; John *et al.*, 2014; Tausta *et al.*, 2014). It turned out that C_4_ photosynthesis is a complex trait and its evolution involved changes in the expression of thousands of genes. Genes encoding the enzymes and transporters of the C_4_ pathway had to be up-regulated and acquired tissue-specific expression. In addition, several other metabolic pathways must also have been regulated differentially in mesophyll and bundle sheath cells to enable this efficient type of photosynthesis including high nitrogen and water use efficiency attributed to C_4_ plants.

It is widely accepted that the development of a photorespiratory CO_2_ pump, often termed C_2_ photosynthesis, was an important intermediate step during the evolution of the C_4_ pathway (Bauwe, 2011; Sage *et al.*, 2012; Heckmann *et al.*, 2013; Williams *et al.*, 2013). The photorespiratory pump is based on the restriction of one of the key photorespiratory enzyme complexes, the glycine decarboxylase complex (GDC), to the bundle sheath cells (Rawsthorne *et al.*, 1988a). Photorespiratory glycine has to move to the bundle sheath for decarboxylation, and CO_2_ is released mainly in this compartment, leading to increased CO_2_ concentrations and allowing RubisCO to work more efficiently (Bauwe, 2011; Heckmann *et al.*, 2013). The photorespiratory pump can lead to a 3-fold enrichment of CO_2_ in the bundle sheath cells (Keerberg *et al.*, 2014). The analysis of C_3_–C_4_ intermediate *Flaveria* species implied that the effect of the photorespiratory pump on C_4_ evolution might be quite direct and provided a mechanistic explanation for how the photorespiratory pump and C_4_ photosynthesis interact (Mallmann *et al.*, 2014). The glycine shuttle induces a nitrogen imbalance between mesophyll and bundle sheath cells, and the introduction of important components of the C_4_ pathway, as well as the C_4_ pathway itself, are highly efficient ways to correct this imbalance. This implies that C_4_ evolution is a metabolic exaptation as the C_4_ pathway developed in the first place to transport nitrogen and was not directly related to improving photosynthetic efficiency (Mallmann *et al.*, 2014). Hence, photorespiration and the cell-specific expression of photorespiratory genes in the mesophyll and bundle sheath cells of C_3_–C_4_ intermediates were of key importance for the evolution of C_4_ photosynthesis.

In the present study, we examined how the expression of photorespiratory genes changed after the transition to true C_4_ photosynthesis. Therefore we analyzed the expression of photosynthetic and photorespiratory genes in the C_4_ grass *Sorghum bicolor* by RNA *in situ* hybridization and transcriptome analysis of isolated mesophyll and bundle sheath fractions. *Sorghum bicolor* is a highly optimized plant species with regard to the C_4_ pathway. Methods for the isolation of mesophyll and bundle sheath cells are available (Wyrich *et al.*, 1998) and its genome is fully sequenced (Paterson *et al.*, 2009), allowing transcriptome analysis with plain high-throughput sequencing as well as with a serial analysis of gene expression (SAGE) approach since the short sequence reads could be directly mapped to the genome or the derived transcriptome sequence (Bräutigam and Gowik, 2010). We determined transcript abundances within our mesophyll and bundle sheath RNA preparations by Illumina sequencing and additionally by SuperSage (Matsumura *et al.*, 2003), a combination of SAGE with next-generation sequencing methods.

We hypothesized that the distribution of photorespiratory gene expression is similar to the enzyme distributions determined previously (Ohnishi and Kanai, 1983; Gardeström *et al.*, 1985; Ohnishi *et al.*, 1985) and that it is comparable in specificity with the distribution of genes related to the C_4_ pathway.

## Materials and methods

### Plant material, RNA isolation, and cDNA synthesis

*Sorghum bicolor* L. Tx430 (Pioneer Hi-Bred, Plainview, TX, USA) was grown on soil (Floraton 1, Floragard, Oldenburg, Germany) in the greenhouse of the Heinrich-Heine University (Düsseldorf, Germany) with supplementary light for 14h per day (~300 μmol m^−2^ s^−1^). For the *in situ* analysis, we harvested the middle thirds of the second leaf from 3-week-old plants and took 2×5mm sections from it. For isolation of mesophyll and bundle sheath RNA, we harvested the upper two-thirds of the second leaf from 10-day-old seedlings. For generation of the cell-specific mRNAs, we separated the bundle sheath and vascular bundles enzymatically from the mesophyll and epidermal cells as described in Wyrich *et al.* (1998). We isolated 15 independent mesophyll and 19 independent bundle sheath samples. Cross-contaminations of the RNA preparations were controlled by dot blot analysis following standard procedures. Five independent mesophyll and bundle sheath preparations were pooled for the SuperSage analysis. For cDNA synthesis and Illumina sequencing, we pooled five other preparations for each tissue. Total RNA from intact *Sorghum* leaves was isolated according to Westhoff *et al.* (1991). Poly(A)^+^ RNA was enriched by two consecutive rounds of oligo(dT) purification with the Oligotex mRNA Midi Kit (Qiagen, Hilden, Germany). cDNA libraries for Illumina sequencing were prepared with the SMARTer PCR cDNA Synthesis Kit (Clontech-Takara Bio Company, Otsu, Japan), with 300ng of poly(A)^+^ RNA as starting material. The purity and integrity of total RNA, poly(A)^+^ RNA, and cDNA were verified spectroscopically with a NanoDrop ND-1000, with the Agilent 2100 Bioanalyzer and by agarose gel electrophoresis.

### SuperSage/Illumina sequencing

The SuperSage analysis was performed by GenXPro Inc. (Frankfurt, Germany) (Matsumura *et al.*, 2003). The mesophyll, bundle sheath, and total cDNA libraries were sequenced each in one lane of an Illumina flow cell with an Illumina Genome Analyser II by GATC Biotech AG (Konstanz, Germany) following standard protocols. The read length was 40bp. The cDNAs were prepared from pooled total RNAs.

### Mapping/statistics

The SuperSage tags as well as the Illumina reads were mapped on the *S. bicolor* transcriptome [version 1.4 (Sbicolor_79_transcript_primaryTranscriptOnly.fa) in the case of the SuperSage tags, and version 3.1 (Sbicolor_313_v3.1.transcript_primaryTranscriptOnly.fa) in the case of the Illumina reads (http://phytozome.jgi.doe.gov)]. The SuperSage tags were mapped with BLAST (Altschul *et al.*, 1990) by GenXPro Inc. Two mismatches were allowed and only tags that were found at least twice were counted. Tag counts were transformed to tags per million (tpm). For the mapping of the Illumina reads, we used BOWTIE (Langmead *et al.*, 2009). The best hit for each Illumina read was retained, and hit counts were then transformed to reads per kilobase and million (RPKM) to normalize for the number of reads available for each cDNA library.

Log2 ratios were calculated and differentially expressed transcripts were called using the R package DEGseq (Wang *et al.*, 2010) on the non-normalized read counts followed by a Bonferroni correction to account for the accumulation of alpha-type errors when conducting multiple pairwise comparisons.

### qRT-PCR

Quantitative real-time PCR (qRT-PCR) followed standard procedures and was performed with an ABI7500 fast Real Time PCR system. The primers were designed to target photorespiratory genes of *S. bicolor* and to generate amplicons of 170bp. The specificity of PCRs was verified by melting curve analysis and agarose gel electrophoresis. To estimate the efficiency of the PCRs, four consecutive 5-fold dilutions of the cDNAs were tested with each primer pair. Only reactions with efficiencies >90% were considered for further analysis. As template we used total RNAs pooled from five independent mesophyll and bundle sheath preparations each, not used for SuperSAGE or Illumina sequencing.

### RNA *in situ* hybridization

The tissue was fixed for 16h in a mixture of 3.7% formaldehyde, 50% ethanol, and 5% acetic acid at 4 °C. Dehydration and embedding was done in the Tissue Processor Leica ASP300S using the following program: 1h in 50% ethanol, 1h in 70% ethanol, 1h in 95% ethanol, 3×1h in 100% ethanol, 2×1h in 100% xylene, 1h in 100% xylene (37 °C), 2×10min in histowax (62 °C), and 20min in histowax (62 °C). Subsequently the samples were embedded in paraffin and cut into 12 µm sections with a microtome.

Probe labeling: for the generation of hybridization probes, the respective cDNAs were amplified by PCR and cloned into pJET1.2/blunt plasmid (Thermo Scientific, St. Leon-Rot, Germany). After linearization of the vector with appropriate restriction enzymes, T7 RNA polymerase was used to generate both sense and antisense probes, which were labeled with digoxigenin (DIG)-labeled UTP using the DIG RNA Labeling kit (Roche, Mannheim, Germany). Subsequently the probes were hydrolyzed to a size of ~150–200 bases.

Pre-hybridization, hybridization, and post-hybridization steps were based on the protocol described by Simon (2002). Only deviations from this protocol are mentioned below. First the sections were dewaxed in Roti^®^-Histol for 10min and rehydrated in a decreasing ethanol concentration series: 2×1min in 100% ethanol, 1min in 95% ethanol, 1min in 85% ethanol, 1min in 50% ethanol, 1min in 30% ethanol, and 1min in ddH_2_O. Afterwards the sections were treated with 10 µg ml^–1^ proteinase K for 30min at 37 °C, post-fixed and acetylated as described by Simon (2002), and finally dehydrated in a reverse order of the ethanol concentration series used before. For the hybridization, 150ng of probe was used for each slide. The sections were incubated for 16h at 50 °C in a humid chamber.

After hybridization, the sections were washed three times in washing buffer (2× SSC, 50% formamide) for 30min at 50 °C and twice in NTE buffer (500mM NaCl, 10mM Tris, 1mM EDTA, pH 8.0) for 5min at 37 °C. After RNase A treatment, the sections were washed again twice in NTE at room temperature for 5min and in washing buffer for 1h at 50 °C.

For immunological detection, all steps were performed on a shaking platform. First the sections were washed in buffer 1 (100mM Tris-HCl pH 7.5, 150mM NaCl) for 5min, before they were incubated in buffer 2 (buffer 1 containing 0.5% blocking reagent; Roche) for 40min. Subsequently they were incubated in buffer 3 (buffer 1 containing 0.3% Triton X-100, 1% normal sheep serum, and sheep anti-DIG–alkaline phosphatase at a dilution of 1:2000) for 2h, after which they were washed four times in buffer 1 containing 0.3% Triton X-100 for 15min. Then the sections were washed in buffer 1 for 5min, incubated in buffer 4 (0.1M Tris-HCl pH 9.5, 0.1M NaCl, and 50mM MgCl_2_) for 5min, and finally stained in buffer 5 [buffer 4 containing 10% polyvinyl alcohol, 0.16mM nitroblue tetrazolium (NBT), and 0.15mM BZIP] in a humid chamber for 12–16h. The reaction was stopped by washing the sections twice in distilled water, after which they were mounted with Entellan^®^ (Merck Millipore, Darmstadt, Germany).

## Results

### Mesophyll and bundle sheath RNAs

**Fig. 1.**
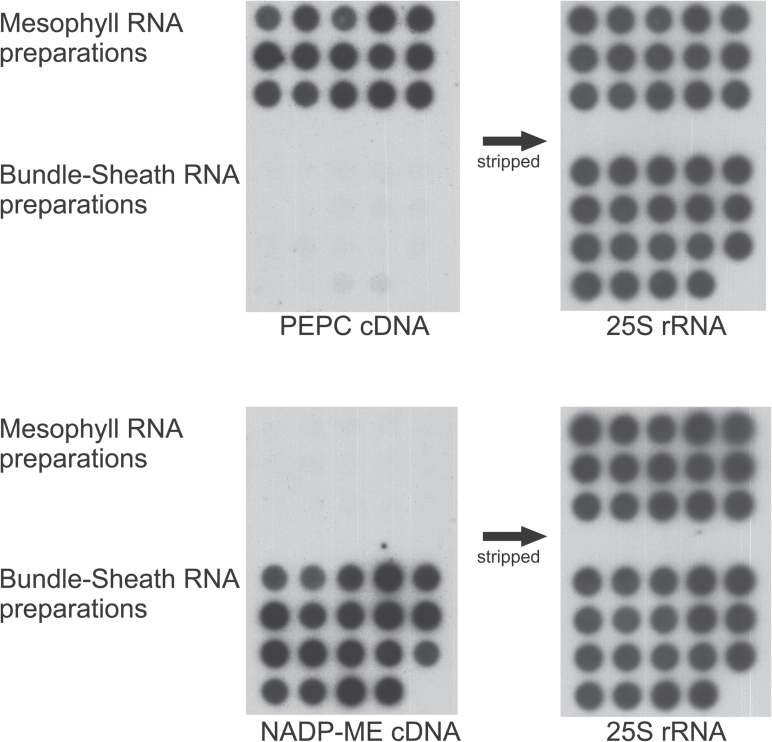
Dot-blot analysis of independent mesophyll and bundle sheath RNA preparations. *Sorghum bicolor* PEPC cDNA, NADP-ME cDNA, and 25S rRNA were used as probes.

Mesophyll and bundle sheath cells of *S. bicolor* for RNA preparations were separated by enzymatic digestion of leaf cell walls as described in Wyrich *et al.* (1998). It has to be considered that the mesophyll fraction also contains epidermis cells whereas the bundle sheath fraction contains all vascular tissues. The cross-contamination of mesophyll and bundle sheath preparations was analyzed by dot blot analysis using a PEPC and an NADP-ME cDNA as hybridization probes (Fig. 1). PEPC is thought to be mesophyll specific in *Sorghum* whereas NADP-ME was shown to be exclusively expressed in bundle sheath cells (Wyrich *et al.*, 1998). Since no signals indicating cross-contamination were visible, it can be assumed that the RNA preparations are pure and that the cross-contamination of mesophyll and bundle sheath RNAs is <5% (Fig. 1).

### Transcriptome analysis via SuperSage and RNA-Seq

To create transcriptome atlases of *Sorghum* bundle sheath and mesophyll tissue, we performed plain Illumina sequencing and a SuperSage analysis. With the SuperSage method, we obtained >6.8×10^6^ tags (total leaf, 1 098 800; mesophyll, 3 349 814; bundle sheath, 2 421 27) that could be assigned to >12 000 (12 937) of the 34 211 predicted *Sorghum* genes, whereas 2327 genes exhibited a significantly different expression between mesophyll and bundle sheath cells (*P*<0.01) (Table 1). With plain Illumina sequencing we produced >36×10^6^ reads (total leaf, 17 704 772; mesophyll, 10 420 446; bundle sheath, 8 695 328) which could be mapped to 23 244 *Sorghum* genes. With this method, we identified 1705 genes as being expressed significantly differentially between mesophyll and bundle sheath cells (*P*<0.01) (Table 1; Supplementary Table S1 at *JXB* online). With the SuperSage approach, we detected far fewer transcripts compared with the plain Illumina sequencing. This is most probably due to limitations of the SuperSage method. A transcript will not be recognized if the cleavage site of the anchoring enzyme, which is needed to produce the DNA fragments used as tags, is not present in the transcript (Matsumura *et al.*, 2003).

**Table 1.** Overview of the SuperSage and RNA-Seq results

	**SuperSage**	**RNA-Seq**
Total reads:	6 870 541	36 820 546
Genes detected (*S. bicolor* 34 211 genes):	12 937	23 244
Percentage:	37	67
Differentially expressed:	2327	1705
Percentage:	6.8	4.9

In total, we were able to detect 12 154 transcripts expressed within the *Sorghum* leaf with at least one read in both experiments, which corresponds to 35% of the total number of transcripts predicted from the *Sorghum* genome sequence (Paterson *et al.*, 2009). A total of 455 (3.7%) of them were more abundant in mesophyll cells and 401 (3.2%) in the bundle sheath in both experiments.

### The enzymatic separation of mesophyll and bundle sheath cells influences gene expression

During the separation of mesophyll and bundle sheath cells by enzymatic digest, the tissue is incubated for up to 2.5h at 25 °C. It is known that this treatment stresses the plant cells and leads to the expression of stress-related genes (Sawers *et al.*, 2007). To account for this problem, we isolated RNA from complete, unstressed *Sorghum* leaves. We assumed that mesophyll and bundle sheath RNA accounts for a comparable fraction of the whole leaf RNA. Based on this premise, we identified 3697 genes within the SuperSage experiment and 3724 genes within the RNA-Seq experiment that were up-regulated >3-fold apparently due to the enzymatic treatment. To test this assumption, we analyzed the representation of Gene Ontology (GO) terms for the up-regulated genes. Indeed, we found an over-representation of GO terms related to stress response among these 3-fold up-regulated genes in the SuperSage as well as in the RNA-Seq experiment (Tables 2, 3). The genes found to be >3-fold up- or down-regulated after enzyme treatment were tagged.

**Table 2.** GO term over-representation analysis of genes up-regulated >3-fold in mesophyll or bundle sheath RNAs compared with total leaf RNA within the Illumina RNA-Seq experiment The 10 most strongly over-represented GO terms are shown. Analysis was performed using the Gene Ontology Consortium database (http://geneontology.org).

**GO term**	**GO name**	***P*-value**
GO:0050896	Response to stimulus	9.40E-17
GO:1901701	Response to oxygen-containing compound	1.57E-13
GO:0042221	Response to chemical	5.65E-12
GO:0001101	Response to acid chemical	5.65E-12
GO:0006950	Response to stress	2.83E-11
GO:0044699	Single-organism process	4.35E-11
GO:0071704	Single-organism cellular process	5.77E-11
GO:0009719	Response to endogenous stimulus	1.20E-10
GO:0071229	Cellular response to acid chemical	2.57E-10
GO:0010033	Response to organic substance	2.79E-10

*P*-values are corrected by the Bonferroni method.

**Table 3.** GO term over-representation analysis of genes up-regulated >3-fold in mesophyll or bundle sheath RNAs compared with total leaf RNA within the SuperSage experiment The 10 most strongly over-represented GO terms are shown. Analysis was performed using the Gene Ontology Consortium database (http://geneontology.org).

**GO term**	**GO name**	***P*-value**
GO:0042221	Response to chemical	3.88E-17
GO:1901700	Response to oxygen-containing compound	1.24E-16
GO:0050896	Response to stimulus	2.02E-16
GO:0009987	Cellular process	8.90E-16
GO:0044237	Cellular metabolic process	1.98E-15
GO:0044699	Single-organism process	1.73E-14
GO:0009628	Response to abiotic stimulus	1.83E-14
GO:0044710	Single-organism metabolic process	1.92E-14
GO:0006950	Response to stress	4.25E-14
GO:0010033	Response to organic substance	4.74E-14

*P*-values are corrected by the Bonferroni method.

### The photorespiratory cycle mainly takes place in the bundle sheath in *S. bicolor*

**Fig. 2.**
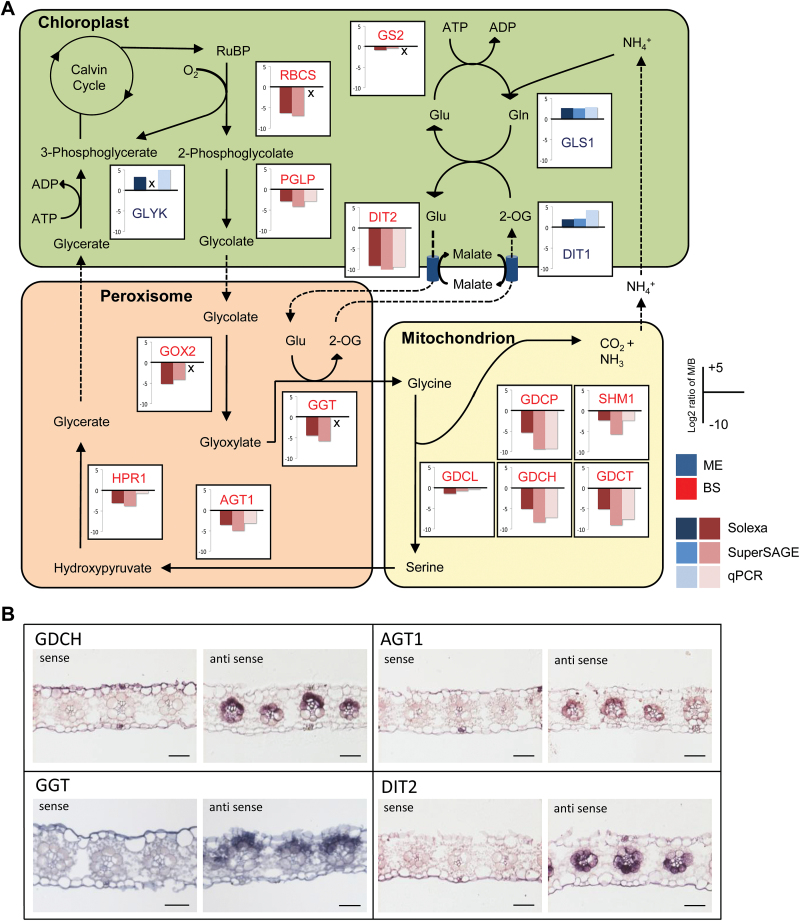
(A) Distribution of photorespiratory genes between mesophyll and bundle sheath cells. Preferential gene expression in the mesophyll and bundle sheath is indicated by blue or red color, respectively. AGT, serine glyoxylate aminotransferase; DIT1+2, dicarboxylate transporter 1+2; GDCP/GDCL/GDCH/GDCT, glycine decarboxylase H, L, P, and T subunit; GGT, glutamate glyoxylate aminotransferase; GLS, glutamate synthase; GLYK, glycerate kinase; GOX2, glycolate oxidase 2; GS, glutamine synthetase; HPR, hydroxypyruvate reductases; PGLP, phosphoglycolate phosphatase; SHM, serine hydroxymethyltransferase; RBCS, ribulose bisphosphate carboxylase/oxygenase small subunit. (B) RNA *in situ* hybridization of *Sorghum bicolor* leaves with probes for transcripts related to photorespiration. Scale bars=50 µm.

It was assumed earlier that in C_4_ plants the photorespiratory pathway is mainly located in the bundle sheath cells since in C_4_ plants, RubisCO, the entry enzyme of photorespiration, is restricted to this cell type (Bauwe, 2011). One exception is glycerate kinase (GLYK), which catalyzes the regeneration of 3-phosphoglycerate (3-PG) and was found to be restricted to the mesophyll cells (Usuda and Edwards, 1980). The present transcriptome analysis largely supports these expectations (Fig. 2; Supplementary Table S2), as do the *in situ* hybridizations (Fig. 2; Supplementary Fig. S1). We detected a strong signal in the bundle sheath for most transcripts of the core photorespiratory pathway with genes that show virtually no expression in the mesophyll and can be seen as bundle sheath specific, such as phosphoglycolate phosphatase (PGLP), glycolate oxidase (GOX), serine hydroxymethyl transferase (SHM), and the H, P, and T subunit of the GDC (Fig. 2; Supplementary Fig. S1). However, there are also genes such as glycine 2-oxoglutarate aminotransferase (GGT) and the GDC L subunit that, although preferentially expressed in the bundle sheath, still seem to be expressed to a certain extent in the mesophyll (Fig. 2; Supplementary Fig. S1). Taken together, this implies that all genes of the core photorespiratory pathway are at least preferentially if not specifically expressed in the bundle sheath, except for GLYK that is expressed to a much higher level in the mesophyll than in the bundle sheath (Fig. 2; Supplementary Table S2). We did not obtain any *in situ* hybridization signal for GLYK. This may be caused by the low absolute expression of the gene observed even in the mesophyll (Supplementary Table S2).

### The transcriptome analysis reveals detailed insight into the C_4_ pathway of *S. bicolor*

**Fig. 3.**
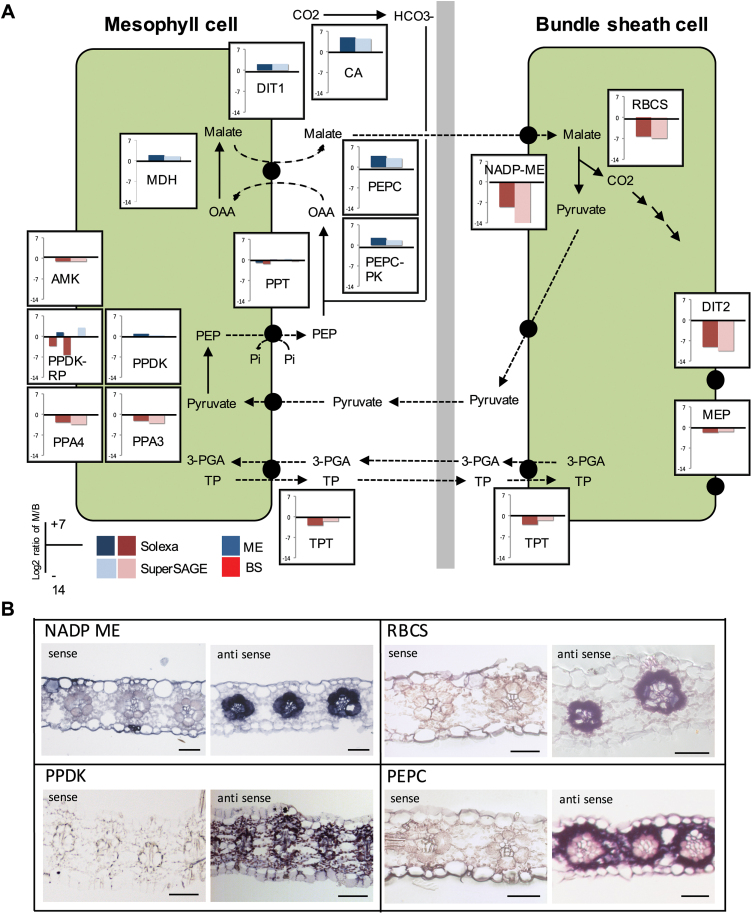
(A) Distribution of C_4_ cycle genes between mesophyll and bundle sheath cells. Preferential gene expression in the mesophyll and bundle sheath is indicated by blue or red color, respectively. AMK, AMP kinase; CA, carbonic anhydrase; DIT1+2, dicarboxylate transporter 1+2; MDH, NADP-dependent malate dehydrogenase; MEP, mesophyll envelope protein; NADP-ME, NADP-dependent malic enzyme; PEPC, phosphoenolpyruvate carboxylase; PEPC-PK, phosphoenolpyruvate carboxylase protein kinase; PPA3+4, pyrophosphorylase 3+4; PPDK, pyruvate phosphate dikinase; PPT, phosphoenolpyruvate phosphate translocator; PPDK-RP, PPDK regulatory protein; RubisCO, ribulose bisphosphate carboxylase/oxygenase; TPT, triosephosphate phosphate translocator. (B) RNA *in situ* hybridization of *Sorghum bicolor* leaves with probes for transcripts related to the C_4_ pathway. Scale bars=50 µm.

*Sorghum bicolor* belongs to the NADP-ME type of C_4_ plants. The genes encoding PEPC, malate dehydrogenase (MDH), or PPDK are expected to be expressed specifically or at least strongly preferentially in the mesophyll in these plants, whereas the genes encoding NADP-ME or RubisCO are bundle sheath specific. The results of our transcriptome analyses are essentially in line with these expectations (Fig. 3; Supplementary Table S3). Although PEPC was found to be expressed preferentially in the mesophyll, as expected, the absolute transcript levels as estimated by the Illumina sequencing appear to be quite low compared with NADP-ME or PPDK. In contrast, PEPC transcript levels turned out to be quite high when determined by the SuperSage method (Supplementary Table S3). If and how we selected against detecting high levels of the PEPC during the Illumina analysis is unclear. We detected virtually no expression of bundle sheath genes such as NADP-ME or RubisCO in the mesophyll, indicating that our mesophyll RNA preparations were not cross-contaminated with bundle sheath RNA (Supplementary Tables S2, S3). The fact that we detected some expression of typical mesophyll genes such as PEPC in the bundle sheath indicates some contamination of our bundle sheath RNA preparation with mesophyll RNA in the range of ~5% (Supplementary Table S3).

Recent results indicate that the classification of the different types of the C_4_ pathway is not as clear-cut as previously thought (Furbank, 2011; Pick *et al.*, 2011; Y. Wang *et al.*, 2014). Maize, which was assumed to be an archetypal NADP-ME-type C_4_ plant, uses in parallel the PEP-CK type pathway to a considerable extent (Wingler *et al.*, 1999; Pick *et al.*, 2011). Interestingly this does not apply for *Sorghum*, although maize and *Sorghum* possess a common C_4_ origin. We did not find a highly expressed PEP-CK gene in bundle sheath cells and no abundantly expressed NAD-ME genes could be detected (Supplementary Table S3). It follows that *Sorghum* instead of maize should be considered as the NADP-ME C_4_ archetype. It was shown earlier that, in contrast to the common textbook models of this pathway, some NADP-ME species use alanine and aspartate as transport metabolites in parallel to malate and pyruvate (Meister *et al.*, 1996; Gowik *et al.*, 2011). We were interested in whether the same is true for *Sorghum*, but the results are inconclusive. While we could identify a highly expressed aspartate aminotransferase (AspAT) gene in mesophyll as well as in bundle sheath cells, we have not found an alanine aminotransferase (AlaAT) that is highly expressed in both cell types. The most highly abundant AlaAT transcript, which belongs to the most abundant transcripts identified in this study, is clearly mesophyll specific. The function of this highly abundant AlaAT in the mesophyll remains unknown. We found another AlaAT gene which was significantly more highly expressed in the bundle sheath compared with the mesophyll (Supplementary Table S3) but, since its overall abundance is much lower, it is unclear if the overall AlaAT transcript abundance in the bundle sheath allows the considerable usage of alanine and aspartate as transport metabolites. The up-regulated AspAT, on the other hand, is predicted to be localized in the chloroplast (TargetP score: 0.968). This in line with other NADP-ME species that synthesize and decarboxylate aspartate in the chloroplasts of mesophyll and bundle sheath cells (Meister *et al.*, 1996; Gowik *et al.*, 2011).

Transcripts related to most of the known transporters thought to be directly involved in the NADP-ME C_4_ pathway, such as the triosephosphate phosphate translocator (TPT), the PEP phosphate translocator (PPT), the dicarboxylate transporter (DIT/DCT/OMT), or the inner chloroplast envelope transporter MEP (Weber and von Caemmerer, 2010) could be identified, and most of them showed high abundance in agreement with their probable role in the C_4_ pathway (Fig. 3; Supplementary Table S3). However, it has to be considered that they did not always show the expected distribution in the two cell types (e.g. the PPT was expected to be mesophyll specific but we also found high amounts of PPT transcripts in the bundle sheath). We could not detect high expression for the BASS2 and the NHD transporter that were shown to catalyze pyruvate transport across the chloroplast membrane in the C_4_ *Flaveria* species (Furumoto *et al.*, 2011). This is in line with earlier results indicating that *Sorghum* uses a proton-dependent pyruvate transporter (Aoki *et al.*, 1992) instead of BASS, which was shown to be a pyruvate–sodium symporter (Furumoto *et al.*, 2011).

While most of the core C_4_ genes are expressed either mesophyll or bundle sheath specifically, as expected, we found that PPDK transcripts are not only highly abundant in the mesophyll, but were also present in respectable amounts in the bundle sheath, with a mesophyll to bundle sheath ratio of only ~1 to 2 (Fig. 3). Along with that, we also found that transcripts related to the PPDK reaction such as pyrophosphatases, AMP kinase, or the PPT exhibit high levels in the bundle sheath cells and are partly even preferentially expressed in the bundle sheath (Supplementary Table S3).

To verify the tissue distribution of selected transcripts, we performed *in situ* hybridizations for typical C_4_ genes such as PEPC, NADP-ME, PPDK, and RBCS (RubisCO small subunit). The obtained results largely support the outcome of the transcriptome analysis using SuperSage or RNA-Seq (compare Fig. 3A and B). *In situ* hybridization confirmed bundle sheath-specific expression for RBCS and NADP-ME, mesophyll-specific expression for the PEPC gene, and the preferential expression in the mesophyll cells of PPDK, with high PPDK transcript levels also in the bundle sheath.

### Expression patterns of genes associated with photorespiration are variable

During photorespiration not only CO_2_, but also nitrogen is released in the mitochondria in the form of NH_3_ that becomes reassimilated in the chloroplasts. In contrast to the core photorespiratory pathway, the genes for nitrogen assimilation and the dedicated transporters do not show a tissue-specific expression pattern. Glutamine synthetase as well as glutamate synthase genes are expressed in mesophyll and bundle sheath cells, but glutamine synthetase is more highly expressed in the bundle sheath, and a ferredoxin-dependent glutamine oxoglutarate aminotransferase (Fd-GOGAT) shows higher transcript abundance in the mesophyll (Fig. 2; Supplementary Table D2).

Only a few transporters involved in the intracellular transport of photorespiratory metabolites are known to date. We could identify two transcripts corresponding to the plastid glycolate glycerate transporter (Pick *et al.*, 2013). Whereas one of the genes appears not to be expressed at all in the *Sorghum* leaf, the other one exhibits high amounts of transcripts in both cell types, but the expression in the bundle sheath is higher than in the mesophyll (Fig. 2; Supplementary Table S2). The mitochondrial transporter BOU, known to be needed for functional photorespiration in *Arabidopsis thaliana* (Eisenhut *et al.*, 2013), appears to be expressed only at a low level in the leaves of the C_4_ plant *Sorghum* and does not show a strong tissue preference (Supplementary Table S2). *Sorghum* contains five genes encoding dicarboxylate transporters (DITs); four of these transporters are classified as DIT2 and one is classified as a DIT1 gene. The DIT1 gene is expressed to moderate levels and clearly is expressed preferentially in the mesophyll. One of the DIT2 genes is highly expressed in the bundle sheath (Fig. 2; Supplementary Table S2). The two transporters are thought to interact in the glutamate–oxoglutarate exchange across the chloroplast membrane during NH_3_ reassimilation (Renne *et al.*, 2003; Bauwe *et al.*, 2010). Additionally the DIT proteins might be involved in the C_4_ cycle of NADP-ME C_4_ species and facilitate the exchange of malate and/or aspartate across the chloroplast membrane (Gowik *et al.*, 2011; Kinoshita *et al.*, 2011), which may explain the highly tissue-preferential expression of these genes in *Sorghum*.

## Discussion

C_4_ photosynthesis mainly evolved to enhance photosynthetic efficiency by avoiding photorespiration. It is widely accepted that an important initial step towards the evolution of C_4_ was the establishment of a photorespiratory CO_2_ pump (Bauwe, 2011; Sage *et al.*, 2012). This was achieved by restricting the activity of a central photorespiratory protein complex, the GDC, to the bundle sheath cells, allowing the release of photorespiratory CO_2_ exclusively in this cell type (Hylton *et al.*, 1988; Rawsthorne *et al.*, 1988b). Finally that was realized by restricting the expression of either single GDC subunit genes or all GDC and SHM genes to the bundle sheath (Morgan *et al.*, 1993). Nevertheless, photorespiration is still essential in C_4_ plants (Zelitch *et al.*, 2008) and we were interested in the tissue-specific expression of photorespiratory genes in the mesophyll and bundle sheath cells of a widely optimized C_4_ species. Therefore we analyzed gene expression in leaves of *S. bicolor* using RNA-Seq on isolated mesophyll and bundle sheath transcripts and RNA *in situ* hybridization.

### Photorespiration is largely confined to the bundle sheath cells in *Sorghum*

In C_4_ plants, photorespiration is reduced to low levels compared with C_3_ plants as a result of concentrating CO_2_ around RubisCO (Hatch, 1987). Using RNA-Seq and SuperSage, we were able to detect the transcripts of all core photorespiratory genes as well as of the genes encoding transporters known to be involved in photorespiration. The vast majority of the core photorespiratory genes are expressed preferentially in the bundle sheath. The only noticeable exceptions are GLYK, which is expressed preferentially in the mesophyll, and the two genes encoding the L subunit of the GDC complex (GDCL), which are nearly equally expressed in both cell types. This largely reflects earlier results from the analysis of mesophyll and bundle sheath transcriptomes and proteomes of the C_4_ grass maize (Li *et al.*, 2010; Majeran *et al.*, 2010; Chang *et al.*, 2012) and studies on the enzyme activities in different C_4_ species (Usuda and Edwards, 1980; Ohnishi and Kanai, 1983; Ohnishi *et al.*, 1985). Since in C_4_ plants RubisCO is missing from the mesophyll cells, no 2-PG can be produced there and 2-PG detoxification in this cell type is no longer necessary. Consequently, the expression of photorespiratory genes was switched off in the mesophyll during C_4_ evolution. The photorespiratory enzymes belong to the most highly abundant proteins in the leaves of C_3_ species (Osborne and Freckleton, 2009; Bauwe, 2011). Accordingly, the decrease in these proteins adds to the reduction of RubisCO in C_4_ plants and contributes to the better nitrogen use efficiency found for C_4_ species (Oaks, 1994; Osborne and Freckleton, 2009).

GDCL is not only part of the GDC but is also connected to other multienzyme complexes such as the pyruvate dehydrogenase complex, the 2-oxoglutarate dehydrogenase complex, and the branched-chained 2-oxoacid dehydrogenase that are not involved in photorespiration and have important functions in general cell metabolism (Millar *et al.*, 1999; Marrott *et al.*, 2014). This explains why the genes encoding GDCL have to stay active in the mesophyll of C_4_ plants. An explanation for the preferential expression of GLYK in the mesophyll is less obvious. In advanced C_4_ species using the NADP-ME pathway, such as maize or *Sorghum*, the activity of photosystem II is greatly reduced in the bundle sheath (Woo *et al.*, 1970; Oswald *et al.*, 1990). This requires the reductive phase of the Calvin–Benson cycle to take place in the mesophyll cells, due to a lack of reducing equivalents in the bundle sheath, and is achieved by a phosphoglycerate–triose phosphate shuttle (Weber and von Caemmerer, 2010). It appears to be more efficient to transfer the photorespiratory glycerate directly to the mesophyll chloroplasts to regenerate 3-PG instead of importing it into the bundle sheath chloroplast for regeneration.

The genes involved in photorespiratory ammonia refixation, glutamine synthetase and glutamate synthase, show different expression patterns in mesophyll and bundle sheath cells. While two glutamine synthetase genes are expressed in both cell types with a bundle sheath preference, Fd-GOGAT is preferentially expressed in the mesophyll. This makes sense in the light of lacking reducing equivalents in the bundle sheath and one can assume that the released ammonia is fixed by glutamine synthetase and the resulting glutamine is partially transferred to the mesophyll to generate glutamate.

The plastidic glycolate glycerate transporter PLGG1 (Pick *et al.*, 2013) is expressed in both cell types. This might be due to the fact that glycolate has to be exported from bundle sheath chloroplasts and glycerate must be imported into the chloroplasts in the mesophyll. It is known that the mitochondrial transporter BOU is essential for photorespiration in *A. thaliana* (Eisenhut *et al.*, 2013). Like PLGG, BOU is expressed in both cell types, but the overall transcript abundance is much lower. Since the specific substrate for the BOU transporter is not known (Eisenhut *et al.*, 2013), one can only speculate about possible functions beside photorespiration.

### Specificity of photorespiratory genes is as variable as that of C_4_ genes

With the transcriptome analysis, we confirmed that *S. bicolor* belongs to the NADP-ME type of C_4_ plants since all participating C_4_ genes (Wang *et al.*, 2009) are expressed in a tissue-preferential manner as expected for the NADP-ME archetype. Recent studies in maize revealed that not only the NADP-ME pathway is operating, but a respectable level of PEP-CK activity, up to 25% of the NADP-ME activity, was also found (Pick *et al.*, 2011). In the leaf transcriptome of *S. bicolor* we could find neither any highly expressed PEP-CK gene nor any significantly expressed NAD-ME gene in the bundle sheath. Taken together, these results indicate that *Sorghum* relies solely on the NADP-ME pathway.

As expected, we found PPDK to be one of the most highly expressed genes in the *Sorghum* leaf. Surprisingly, the transcriptome analysis indicated that PPDK transcripts are not restricted to the mesophyll but are also found in high amounts in the bundle sheath, with a mesophyll to bundle sheath ratio of only ~1 to 2 (Fig. 3). We confirmed that the analysis detects only the gene encoding the chloroplast-targeted PPDK isoform and indeed the gene encoding the cytosolic isoform showed quite low expression in *Sorghum* leaves. Also the RNA *in situ* analysis indicates high amounts of PPDK transcripts in the bundle sheath cells (Fig. 3B). Since this analysis is strictly independent of the transcriptome analysis, it must be considered that *Sorghum* contains considerable amounts of PPDK in its bundle sheath cells. This is in contrast to the analysis of mesophyll and bundle sheath cells of maize or *S. viridis* where PPDK transcripts were found to be five and 20 times more abundant in the mesophyll than in the bundle sheath, respectively (Chang *et al.*, 2012; John *et al.*, 2014). Very similar patterns were also found for the transcripts of genes that functionally interact with PPDK such as the PPDK regulatory proteins, plastid-localized pyrophosphatases, an AMP kinase, and the plastid PEP translocator PPT (Fig. 3; Supplementary Table S3). For all these genes, we found considerable amounts of transcripts in the bundle sheath preparations that were often even higher than in the mesophyll. The most parsimonious explanation is that *Sorghum* is capable of regenerating substantial amounts of PEP in the bundle sheath cells. The existence of plants using extensively the PEP-CK type of the C_4_ pathway shows that PEP can serve as a transport metabolite in the C_4_ cycle. Due to up-regulation of photosystem I and cyclic electron transport in the bundle sheath chloroplasts (Supplementary Table S1; Kubicki *et al.*, 1994, 1996), *Sorghum* potentially produces high amounts of ATP in this compartment that can be used for PEP regeneration. By regenerating PEP in the bundle sheath chloroplasts, the number of transport processes would be reduced since PEP can be exported by PPT and diffuse into the mesophyll where it could be carboxylated by PEPC in the cytosol.

All in all, it appears that the degree of cell specificity is quite comparable for photorespiratory and C_4_ cycle genes. While most of the genes encoding core pathway enzymes are expressed in a highly cell type-specific manner, exceptions are the PPDK in the case of the C_4_ cycle and GDCL in the case of photorespiration. This is notable since tissue specificity for C_4_ enzymes such as PEPC or NADP-ME is necessary to avoid futile cycles and ensure the efficiency of the pathway, whereas tissue-specific expression of most photorespiratory genes has to be seen as optimization that saves nitrogen. The expression of auxiliary genes of both pathways was found to be not very tissue specific. This might be due to additional roles of the encoded protein in other important pathways as can be envisaged for the genes involved in primary nitrogen and amino acid metabolism.

### Evolutionary aspects of restricting photorespiration to the bundle sheath

As discussed above, photorespiration was important for the evolution of C_4_ photosynthesis in different ways. The avoidance of photorespiration was one of the driving forces towards C_4_ photosynthesis, and the establishment of a photorespiratory pump was an important intermediate step during C_4_ evolution (Bauwe, 2011; Sage *et al.*, 2012). The reduction and exclusion of the majority of photorespiratory reactions from the mesophyll represents an optimization and enhances the nitrogen use efficiency. This optimization could only happen after the implementation of a fully functional C_4_ pathway and the complete down-regulation of RubisCO in the mesophyll since the oxygenase reaction of RubisCO would be fatal without PGLP and GOX activity present in the same compartment. This has a further implication for C_4_ evolution: once PGLP and GOX are switched off in the mesophyll, the reintroduction of RubisCO into this compartment would be detrimental. Once these photorespiratory reactions are gone from the mesophyll due to optimization, a reversal from C_4_ to C_3_ photosynthesis becomes impossible.

## Supplementary data

Supplementary data are available at *JXB* online.

Table S1. Excel worksheet providing quantitative information for all reads and all SuperSage tags mapped onto the reference transcriptome from *Sorghum bicolor*.

Table S2. Transcript abundance of genes related to photorespiration

Table S3. Transcript abundance of C_4_ cycle genes and C_4_-related transporters.

Table S4. Gene-specific primers used for qPCR and RNA *in situ* analysis.

Figure S1, RNA *in situ* hybridization of *Sorghum bicolor* leaves with probes for transcripts related to photorespiration

Supplementary Data
