# Supplementary material for: Most photorespiratory genes are preferentially expressed in the bundle sheath cells of the C4 grass Sorghum bicolor
Source: J Exp Bot. 2016 Mar 14;67(10):3053–64. doi: 10.1093/jxb/erw041 (PMC4867894; doi:10.1093/jxb/erw041)
Supplement: Supplementary Data [file supp_67_10_3053__index.html]

Most photorespiratory genes are preferentially expressed in the bundle sheath cells of the C4 grass Sorghum bicolor — Most photorespiratory genes are preferentially expressed in the bundle sheath cells of the C4 grass Sorghum bicolor — Supplementary Data 

# Most photorespiratory genes are preferentially expressed in the bundle sheath cells of the C4 grass *Sorghum bicolor*

## Supplementary Data

Data files

- Supplementary\_table\_S1.xlsx - Supplementary Data
- supplementary\_tables\_S2\_S4\_figure\_S1.pdf - Supplementary Data
