# Supplementary material for: Most photorespiratory genes are preferentially expressed in the bundle sheath cells of the C4 grass Sorghum bicolor
Source: J Exp Bot. 2016 Mar 14;67(10):3053–64. doi: 10.1093/jxb/erw041 (PMC4867894; doi:10.1093/jxb/erw041)

Most photorespiratory genes are preferentially expressed in the bundle sheath cells of the C4 grass *Sorghum bicolor*

Florian Döring, Monika Streubel, Andrea Bräutigam and Udo Gowik

Supplementary Data

Supplemental Table 1 Excel worksheet providing quantitative information for all reads and all SuperSage tags mapped onto the reference transcriptome from *Sorghum bicolor*. (not included)

Supplemental Table 2 Transcript abundance of genes related to photorespiration

Supplemental Table 3 Transcript abundance of C4 cycle genes and C4-related transporters.

Supplemental Table 4 Gene-specific primers used for qPCR and RNA *in situ* analysis. F: forward primer; R: reverse primer.

Supplemental Figure 1 RNA *in situ* hybridization of *Sorghum bicolor* leaves with probes for transcripts related to photorespiration

Supplemental Table 2 Transcript abundance of genes related to photorespiration

		Illumina Sequencing						SuperSage					
locusName	gene-symbol	rpmk total leaf	rpmk M	rpmk B	log2 M/B	P-value	3 fold change	tpm total leaf	tpm M	tpm B	log2 M/B	P-value	3 fold change
**Chloroplast**													
Sobic.005G042000	RBCS1A	4233,815	293,974	22898,380	-6,283	0	no	9570,651	137,152	17292,959	-6,978	0	no
Sobic.006G130300	PGLP1	25,734	33,785	253,103	-2,905	3,27023E-37	up	194,911	23,307	416,688	-4,160	2,0241E-88	no
Sobic.001G007000	ATPK5,PGLP2	52,506	11,795	33,562	-1,509	1	no	1,822	2,092	0,413	2,339	1	no
Sobic.009G004100	GLYK	27,647	42,375	4,273	3,310	6,27671E-05	no	0,000	0,000	0,000	NA	NA	no
Sobic.003G259200	GLYK	1,223	1,230	0,609	1,014	1	no	0,000	0,000	0,000	NA	NA	no
**Peroxisome**													
Sobic.001G065600	GOX	14,322	31,815	10,971	1,536	1	no	0,000	0,598	2,894	-2,276	1	up
Sobic.006G220600	GOX	57,505	14,972	4,153	1,850	1	down	1,822	5,080	6,201	-0,288	1	no
Sobic.006G220500	GOX	1,415	2,871	0,000	NA	NA	no	0,911	2,988	0,413	2,854	1	no
Sobic.002G036000	GOX	138,160	3,326	118,320	-5,153	1,83619E-24	no	376,159	57,968	1016,504	-4,132	1,3907E-218	no
Sobic.002G374650	GOX	0,132	0,000	0,296	NA	NA	no	0,000	0,000	0,000	NA	NA	no
Sobic.002G374700	GOX	3,102	0,698	2,032	-1,542	1	no	0,000	0,000	0,000	NA	NA	no
Sobic.002G006600	AOAT1,GGAT1,GGT1	287,781	59,842	1301,550	-4,443	4,6543E-281	no	183,981	11,355	634,953	-5,805	3,2558E-149	no
Sobic.007G213700	AGT,AGT1,SGAT	15,175	2,505	29,156	-3,541	0,017164041	no	325,154	34,363	1077,270	-4,970	3,9618E-250	no
Sobic.004G001300	HPR	730,797	128,514	1080,376	-3,072	2,8341E-177	no	900,778	95,319	1310,004	-3,781	1,9468E-267	no
**Mitochondria**													
Sobic.004G055700	GDCH	3,514	0,000	4,257	NA	NA	no	3,643	0,299	11,575	-5,276	1	no
Sobic.008G062500	GDCH	108,391	6,994	236,982	-5,082	1,2377E-51	no	147,549	0,896	293,087	-8,353	4,09372E-57	no
Sobic.006G220800	GDCT	9,135	1,117	38,370	-5,102	7,05627E-06	no	67,399	0,299	153,778	-9,007	2,55099E-26	no
Sobic.003G152900	GDCL,mtLPD1	21,060	26,198	69,466	-1,407	0,376737044	no	71,953	140,439	233,560	-0,734	0,010363406	no
Sobic.009G054600	GDCL,mtLPD1	6,972	2,338	10,766	-2,203	1	no	15,484	15,239	26,043	-0,773	1	no
Sobic.008G039900	GDCP,GLDP2	124,436	24,931	1013,739	-5,346	6,7786E-232	up	407,126	1,793	1186,816	-9,371	4,9493E-211	no
Sobic.001G097100	SHM1,SHMT1,STM	110,030	48,230	239,734	-2,313	1,61387E-26	no	92,901	4,482	235,627	-5,716	1,00358E-53	no
Sobic.008G144800	SHM4	17,807	1,419	35,486	-4,644	5,96822E-05	no	27,324	3,885	81,023	-4,383	2,22493E-15	no
Sobic.005G113300	SHM4	13,718	5,460	33,354	-2,611	0,118928169	no	79,239	27,191	103,345	-1,926	7,26477E-08	no

Sobic.003G373600	SHM7	27,429	7,661	30,444	-1,991	1	no	3,643	1,195	1,654	-0,468	1	no
**NH_3 fixation**													
Sobic.001G451500	GLN1;1,GSR 1	26,844	52,994	87,376	-0,721	1	no	79,239	338,548	306,315	0,144	1	up
Sobic.004G247000	GLN1;1,GSR 1	72,527	39,107	68,080	-0,800	1	no	1203,162	151,495	729,617	-2,268	3,10968E-90	no
Sobic.006G249400	GLN2,GS2	72,433	106,444	182,593	-0,779	0,554868248	no	418,967	196,316	249,269	-0,345	1	no
Sobic.001G116400	GSR2	5,136	10,446	2,302	2,182	1	no	20,038	13,148	43,405	-1,723	0,800326939	no
Sobic.002G402700	FD-GOGAT,GLS1,GLU1,GLUS	256,269	389,295	62,028	2,650	6,66152E-56	no	751,407	441,934	73,169	2,595	1,23715E-58	no
**Transport**													
Sobic.001G288900	BOU	0,453	3,261	1,016	1,682	1	up	66,488	60,060	45,885	0,388	1	no
Sobic.007G226800	DCT,DIT2.1	3,018	0,290	5,829	-4,331	1	no	0,000	0,000	0,827	NA	NA	up
Sobic.004G035500	DCT,DIT2.1	79,690	1,917	1053,286	-9,102	1,5008E-190	up	481,811	0,598	849,498	-10,473	1,1487E-127	no
Sobic.002G233700	DCT,DIT2.1	28,701	21,046	10,628	0,986	1	no	10,930	2,988	0,413	2,854	1	no
Sobic.007G226700	DCT,DIT2.1	0,061	0,064	0,411	-2,687	1	up	0,000	0,000	0,000	NA	NA	no
Sobic.008G112300	DiT1	19,738	25,761	6,547	1,976	1	no	542,835	177,192	40,511	2,129	1,63389E-16	no
Sobic.001G283100	PLGG	529,288	420,125	940,388	-1,162	2,88768E-39	no	567,426	238,746	367,495	-0,622	0,000697181	no
Sobic.001G283000	PLGG	18,507	17,456	29,825	-0,773	1	no	0,000	0,000	0,000	NA	NA	no

Supplemental Table 3 Transcript abundance of C4 cycle genes and C4-related transporters

		Illumina Sequencing						SuperSage					
locusName	gene-symbol	rpmk total leaf	rpmk M	rpmk B	log2 M/B	P-value	3 fold change	tpm total leaf	tpm M	tpm B	log2 M/B	P-value	3 fold change
**C4 NADP-ME**													
Sobic.003G234200	BCA4,CA4	317,888	606,438	20,134	4,913	9,2011E-139	no	1834,345	3579,400	157,912	4,503	9,2011E-139	no
Sobic.003G234400	BCA4,CA4	27,721	133,032	2,186	5,927	5,48218E-29	no	1799,734	1817,340	73,582	4,626	5,48218E-29	no
Sobic.003G234600	BCA4,CA4	0,671	0,654	0,301	1,121	1	no	0,000	0,000	0,000	NA	1	no
Sobic.003G234500	BETA CA2,CA18,CA2	8,218	34,696	2,554	3,764	0,000384212	no	0,000	2,092	0,000	NA	0,000384212	down
Sobic.010G160700	ATPPC2,PPC2	201,204	182,626	11,960	3,933	8,96566E-36	no	10943,221	9058,013	1062,802	3,091	8,96566E-36	no
Sobic.007G137600	MDH	72,086	387,252	102,225	1,922	4,06286E-37	up	67,399	138,945	47,539	1,547	4,06286E-37	no
Sobic.007G166300	MDH	165,925	368,467	19,380	4,249	1,92794E-78	no	690,384	547,712	20,256	4,757	1,92794E-78	no
Sobic.003G036200	NADP-ME4	1882,793	35,126	12952,277	-8,526	0	up	5182,433	0,299	11702,811	-15,257	0	no
Sobic.003G036000	NADP-ME4	251,720	19,124	1814,836	-6,568	0	up	0,000	0,000	0,000	NA	0	no
Sobic.009G108700	NADP-ME4	123,265	20,356	669,038	-5,039	3,0089E-150	no	0,000	0,000	0,000	NA	3,0089E-150	no
Sobic.009G132900	PPDK	5343,046	8768,331	4789,007	0,873	4,9659E-279	no	6289,051	5985,388	4767,108	0,328	4,9659E-279	no
Sobic.001G326900	PPDK	4,887	5,218	4,856	0,104	1	no	0,000	0,000	0,000	NA	1	no
Sobic.004G331700	AAT3,ASP5,ATAAT1	672,110	397,132	195,499	1,022	1,87001E-13	no	19,127	31,076	6,201	2,325	1,87001E-13	no
Sobic.002G375800	ALAAT2	27,885	9,611	49,477	-2,364	0,003286999	no	32,789	53,785	178,167	-1,728	0,003286999	down
Sobic.001G260800	ALAAT2	3048,522	5066,782	52,774	6,585	0	no	689,473	522,612	31,004	4,075	0	no
Sobic.001G260701	ALAAT2	0,541	0,000	0,520	NA	1	no	0,000	0,000	0,000	NA	1	no
**C4 other**													
Sobic.009G240700	mMDH1	19,529	5,444	18,960	-1,800	1	no	79,239	46,016	99,625	-1,114	1	no
Sobic.003G238500	mMDH2	30,559	0,953	8,624	-3,177	1	down	15,484	6,574	12,815	-0,963	1	no
Sobic.002G309400	NAD-ME1	21,993	34,710	32,878	0,078	1	no	0,000	0,000	0,000	NA	1	no
Sobic.001G201700	NAD-ME2	13,438	17,097	33,952	-0,990	1	no	6,376	25,996	12,402	1,068	1	no
Sobic.001G432800	PCK1,PEPCK	7,651	1,782	8,705	-2,288	1	no	0,000	0,000	0,000	NA	1	no
**transport**													
Sobic.003G002300	APE2,TPT	1082,659	523,077	3903,597	-2,900	0	no	4902,818	1623,414	4609,610	-1,506	0	no
Sobic.009G088200	APE2,TPT	2,637	1,168	0,358	1,706	1	down	0,000	0,000	0,000	NA	1	no

		Illumina Sequencing						SuperSage					
locusName	gene-symbol	rpmk total leaf	rpmk M	rpmk B	log2 M/B	P-value	3 fold change	tpm total leaf	tpm M	tpm B	log2 M/B	P-value	3 fold change
Sobic.009G062500	CUE1,PPT	22,320	8,522	8,326	0,034	1	no	0,000	0,299	0,413	-0,468	1	down
Sobic.002G159900	CUE1,PPT	4,861	0,312	2,011	-2,687	1	down	3,643	5,080	5,374	-0,081	1	no
Sobic.003G050800	CUE1,PPT	33,603	35,119	67,603	-0,945	1	no	142,084	175,997	188,088	-0,096	1	no
Sobic.004G353100	CUE1,PPT	194,165	152,628	378,450	-1,310	1,01749E-17	no	986,393	880,283	695,720	0,339	1,01749E-17	no
Sobic.004G165000	BASS	16,480	9,040	21,089	-1,222	1	no	2,732	2,988	3,307	-0,146	1	no
Sobic.009G237100	BASS	61,350	66,195	16,752	1,982	0,000404007	no	61,934	46,315	9,094	2,348	0,000404007	no
Sobic.003G236800	BASS	3,711	1,192	0,560	1,091	1	down	0,000	0,000	0,000	NA	1	no
Sobic.002G141900	NHD1	15,979	3,477	12,055	-1,794	1	no	3,643	1,195	8,681	-2,861	1	no
Sobic.007G160200	DIC2	0,221	0,000	2,410	NA	1	up	363,408	158,367	304,662	-0,944	1	no
Sobic.003G431900	MEP	95,691	1,197	1053,861	-9,783	8,0009E-174	up	0,9108	0	2,4803	NA	NA	no
Sobic.009G124000	MEP	9,230	2,913	2,666	0,128	1	down	13,6619	2,0916	7,4409	-1,830870155	1	no
Sobic.001G000800	MEP	85,110	73,701	241,883	-1,715	5,85604E-17	no	143,9059	56,1755	153,7776	-1,452832403	5,43622E-08	no
**regultation**													
Sobic.004G219900	ATPPCK1,PPCK1	0,671	6,038	1,041	2,536	1	up	19,127	57,371	16,949	1,759	1	no
Sobic.004G338000	ATPPCK1,PPCK1	0,203	0,000	0,000	NA	1	no	2,732	3,586	2,894	0,309	1	no
Sobic.006G148300	ATPPCK1,PPCK1	0,211	2,960	0,068	5,453	1	up	10,930	2,988	18,602	-2,638	1	no
Sobic.002G324400	PPDK RP1	66,935	63,692	24,359	1,387	0,426463451	no	399,840	319,723	42,165	2,923	0,426463451	no
Sobic.002G324500	PPDK RP1	57,217	4,745	385,412	-6,344	1,94619E-86	up	0,000	0,000	0,000	NA	1,94619E-86	no
Sobic.002G324700	PPDK RP1	13,524	2,707	25,811	-3,253	0,172839961	no	0,000	0,000	0,000	NA	0,172839961	no
**PPDK associated**													
Sobic.007G113600	AMK	90,606	78,516	143,023	-0,865	0,929230866	no	40,986	39,741	58,287	-0,553	0,929230866	no
Sobic.007G009200	AMK	432,031	419,019	858,267	-1,034	9,65255E-29	no	2337,104	732,374	1501,812	-1,036	9,65255E-29	no
Sobic.001G252900	PPa1	0,262	0,000	0,440	NA	1	no	0,000	0,000	0,000	NA	1	no
Sobic.006G276700	PPa3	547,808	75,042	330,830	-2,140	6,76081E-34	no	71,953	16,434	132,282	-3,009	6,76081E-34	no
Sobic.003G367650	PPa3	1,557	31,622	1,903	4,055	0,000817402	up	0,000	0,000	0,000	NA	0,000817402	no
Sobic.009G152600	PPa3	1,626	5,503	0,503	3,453	1	no	0,000	0,000	0,000	NA	1	no
Sobic.004G268000	PPa4	249,840	108,758	699,846	-2,686	6,3416E-99	no	269,596	57,670	627,512	-3,444	6,3416E-99	no

		Illumina Sequencing						SuperSage					
locusName	gene-symbol	rpmk total leaf	rpmk M	rpmk B	log2 M/B	P-value	3 fold change	tpm total leaf	tpm M	tpm B	log2 M/B	P-value	3 fold change
Sobic.009G016000	PPa4	453,310	658,176	1936,839	-1,557	1,0496E-134	no	249,558	223,208	212,891	0,068	1,0496E-134	no
Sobic.004G311100	PPa6	93,319	103,226	203,877	-0,982	0,000738658	no	1515,566	1132,476	1333,567	-0,236	0,000738658	no

Supplemental Table 4 Gene-specific primers used for qPCR and RNA *in situ* analysis. F: forward primer; R: reverse primer.

In Situ

Primer designation	Sequence	Orientation
IS-AGT1	TCGACTGGAAGGACTACCTCA	F
IS-AGT1	AATCTGGGCAGGGCTATACA	R
IS-DIT1	GTGTCTGCAGCAATTCTTGG	F
IS-DIT1	GAGAACGATCCCCAAGATCC	R
IS-DIT2	TGGGATACATTGGCTTGGTT	F
IS-DIT2	TAGAGATTGGTCGCGCTTTT	R
IS-GDCH	GCCTTTGCCTTGTCAATCTC	F
IS-GDCH	CATGGGGATTTTAAGAATCTGG	R
IS-GDCL	ACCACCTGCATCGAGAAGAG	F
IS-GDCL	ATGGTCTGCTCACCACCAG	R
IS-GDCP	CAAGCCCTGGTTTTATTGGA	F
IS-GDCP	CTGCAGTGTGCAGATGAGGT	R
IS-GDCT	CCACATCTACCTCGTCGTCA	F
IS-GDCT	CGAGGGCCTGTAGTACTTGG	R
IS-GGT	TGCCCTATTCCACAGTACCC	F
IS-GGT	ACAACAATCCCAGTGGCTTC	R
IS-GLS1	CAAACTGGACAGGTGCTTGA	F
IS-GLS1	TGGCACCATATCCAATCAGA	R
IS-GLYK	ATCGGCTACACCGATGAGAA	F
IS-GLYK	TCCAGGTTTTCCATCTGCTC	R
IS-GOX2	ACAGGAAAGTGGTGGAGCAG	F
IS-GOX2	ACTCGGTGATGATGTGGTTG	R
IS-GS2	GGTTGCTTTGCTGAAGGTCT	F
IS-GS2	CCTGTGCTTGACCCATCATA	R
IS-HPR1	GGTGACCCTCCCATCTACTTTAC	F
IS-HPR1	AGCAGCCTATATGCACATGTTTT	R
IS-ME	ATCAGTGGTCCTTGCAGGTT	F
IS-ME	GCATATGCCACCAGGTCTTT	R
IS-PEPC	AAGTACGGCGTGAAGCTGAC	F
IS-PEPC	GGGTGGTGATGTAGGGATTG	R
IS-PGLP	CCGTCGAGACCTTCATCTTC	F
IS-PGLP	AATCTGGCTGGATCGAGTTG	R
IS-PPDK	GAAGCTGTATGGCGAGTTCC	F
IS-PPDK	CATTCAGCGATAGCCACTCA	R
IS-RBCS	TTCCAGGGTCTCAAGTCCAC	F
IS-RBCS	ACCAGAGCAAACTCCAATGC	R
IS-SHM1	GCGACCTCCATCTACTTCCA	F
IS-SHM1	CACGGCTTGGTGAAGGTACT	R

qPCR

Primer designation	Sequence	Orientation
qPCR-ACTIN	CGTTTATTTGCATCGGACCT	F
qPCR-ACTIN	TAGCACCAGCACAATCCAAG	R
qPCR-AGT1	CGACTACGACGACGAATGG	F
qPCR-AGT1	GACCAACAGCTGAGTGTGGA	R
qPCR-DIT1	ACTATGGCATTGGCTCTGCT	F
qPCR-DIT1	TCGCAGTTAATGTGGTGCTC	R
qPCR-DIT2	TGGAGCTGGGTATCTTGACC	F
qPCR-DIT2	GCTGCTGTCATCACTTCCAA	R
qPCR-G6PI	ACCGGCCATCACTAAGTTTG	F
qPCR-G6PI	ATGAAGCCCTGAACTGCAAC	R
qPCR-GDCH	CTGATCAACACGAGCCCTTAC	F
qPCR-GDCH	ACTGCGTCATAAGCATGCAC	R
qPCR-GDCL	AAGGTGATCGCAGAGAAGGA	F
qPCR-GDCL	GAGGGCTTCGCTCACTGTAG	R
qPCR-GDCP	CGACTTGGGCAACCTGTTAT	F
qPCR-GDCP	TGCTGAGACAGGAATGATGC	R
qPCR-GDCT	CGCTATGGGCTACGTGAAAT	F
qPCR-GDCT	GCAACAAGAGAAATGCAAAGC	R
qPCR-GGT	GGTTCTGAGCCCTGATGTGT	F
qPCR-GGT	AATGTCAAATGGGCAGAAGC	R
qPCR-GLS1	GCCAGGACGTCCACACTAAT	F
qPCR-GLS1	CTTGGCAATCACACCTGCTA	R
qPCR-GLYK	GATCCACAGCTTGAGGTGGT	F
qPCR-GLYK	TCCAGGTTTTCCATCTGCTC	R
qPCR-GOX2	CGTTCCTTCTCTTCATCATTCAGA	F
qPCR-GOX2	CAACAAGGAACCAAGCCAGTTAC	R
qPCR-GS2	CACAGGTGAGTTGAATCCGC	F
qPCR-GS2	TCACCACGGAATGCAGCAG	R
qPCR-HPR1	CAAAGCTTTAGGAGCCAACG	F
qPCR-HPR1	TTATACCGGAACCCAAGCTG	R
qPCR-PFK	GGTGCTGGACAGGATCTCAT	F
qPCR-PFK	AGGTCGGGTCGATGTACTTG	R
qPCR-PGLP	GGCAAAGAAGTTCGGAATCA	F
qPCR-PGLP	AATCTGGCTGGATCGAGTTG	R
qPCR-PPDK	AGTTGAGAACTGCAACATAC	F
qPCR-PPDK	CTGGTTCTACCATCTTAATC	R
qPCR-RPN	CAATGCCTCAAGCTCAAACA	F
qPCR-RPN	TCAGTCACAGCTGCAGATCC	R
qPCR-SHM1	GAGCAAGGGCTACAAGTTGG	F
qPCR-SHM1	GGTGTCATTGCACTGCTGTC	R

Supplemental Figure 1 RNA *in situ* hybridization of *Sorghum bicolor* leaves with probes for transcripts related to photorespiration

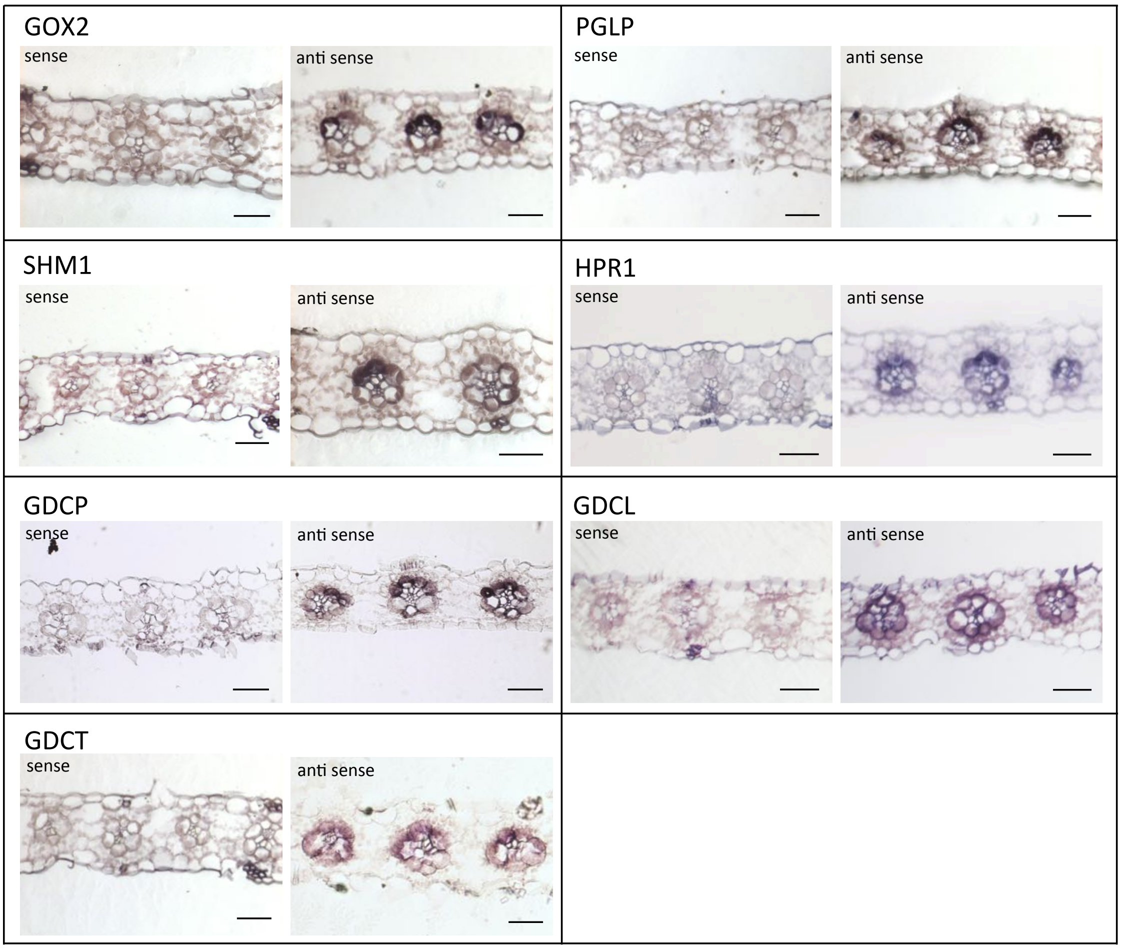

Supplement: Supplementary Data [file supp_erw041_supplementary_tables_S2_S4_figure_S1.pdf]
